# Association between Neck/Shoulder Pain and Trapezius Muscle Tenderness in Office Workers

**DOI:** 10.1155/2014/352735

**Published:** 2014-03-27

**Authors:** Mikkel Brandt, Emil Sundstrup, Markus D. Jakobsen, Kenneth Jay, Juan C. Colado, Yuling Wang, Mette K. Zebis, Lars L. Andersen

**Affiliations:** ^1^National Research Centre for the Working Environment, Lersø Parkalle 105, 2100 Copenhagen Ø, Denmark; ^2^Research Group in Sport and Health, Laboratory of Physical Activity and Health, University of Valencia, 46010 Valencia, Spain; ^3^Department of Rehabilitation Medicine, The Sixth Affiliated Hospital of Sun Yat-sen University, Guangzhou 510000, China; ^4^Metropolitan University College, Tagensvej 18, 2200 Copenhagen N, Denmark

## Abstract

*Background*. Neck/shoulder pain is a common musculoskeletal disorder among adults. The pain is often assumed to be related to muscular tenderness rather than serious chronic disease. *Aim*. To determine the association between neck/shoulder pain intensity and trapezius muscle tenderness in office workers. *Methods*. 653 employees from two large office workplaces in Copenhagen, Denmark, replied to a questionnaire on health and working conditions (mean: age 43 years, body mass index 24 kg*·*m^−2^, computer use 90% of work time, 73% women). Respondents rated intensity of neck/shoulder pain during the previous three months on a scale of 0–10 and palpable tenderness of the upper trapezius muscle on a scale of “no tenderness,” “some tenderness,” or “severe tenderness.” Odds ratios for tenderness as a function of neck/shoulder pain intensity were determined using cumulative logistic regression controlled for age, gender, and chronic disease. *Results*. The prevalence of “no,” “some,” and “severe” tenderness of the trapezius muscle was 18%, 59%, and 23% in women and 51%, 42%, and 7% in men, respectively (chi-square, *P* < 0.0001). Participants with “no,” “some,” and “severe” tenderness of the trapezius muscle, respectively, rated their neck/shoulder pain intensity to 1.5 (SD 1.6), 3.8 (SD 2.0), and 5.7 (SD 1.9) for women and 1.4 (SD 1.4), 3.1 (SD 2.2), and 5.1 (SD 1.7) for men. For every unit increase in neck/shoulder pain intensity, the OR for one unit increase in trapezius tenderness was 1.86 (95% confidence interval 1.70 to 2.04). *Conclusion*. In office workers, a strong association between perceived neck/shoulder pain intensity and trapezius muscle tenderness exists. The present study provides reference values of pain intensity among office workers with no, some, and severe tenderness of the trapezius muscle.

## 1. Introduction

Approximately a third of working-age adults are regularly bothered by neck pain [1], and every other office worker experiences neck/shoulder pain on a weekly basis [2, 3]. Musculoskeletal disorders of the neck and shoulder in office workers are likely influenced by prolonged static working positions [4], leading to continuous activity of low-threshold motor units, reduced local blood flow, accumulation of Ca^2+^, and other homeostatic changes in the active muscle fibers [5, 6]. Thus, pain symptoms appear to worsen during prolonged static muscle activity and repetitive job tasks [7, 8]. The associated costs are enormous, as white-collar workers with neck/shoulder pain have a 35% increased risk of long-term sickness absence [9].

Many people experience soreness of the neck/shoulder muscles after prolonged computer work. The soreness presents in different neck/shoulder muscles, for example, the trapezius, levator scapulae, neck extensors, and infraspinatus [10, 11]. For some people the soreness and pain aggravates over time and becomes chronic. Clinical research has confirmed that the most common type of neck/shoulder pain in computer workers is associated with tenderness of the muscles, that is, myalgia [12]. In a small sample of elderly computer workers with neck/shoulder pain 38% had trapezius myalgia [12]. However, a more detailed relationship between neck/shoulder pain intensity and trapezius muscle tenderness remains unclear.

The aim of the present study is to determine associations between neck/shoulder pain intensity and trapezius muscle tenderness in office workers.

## 2. Methods

### 2.1. Study Design and Participants

The present questionnaire survey was conducted in June 2009 as part of a randomised controlled trial [13], which was approved by the local ethical committee of Copenhagen and Frederiksberg (HC2008103). The study was registered at the Danish Data Protection Agency (registration number 2009-54-0737). The questionnaire on health and working conditions went out to 1094 employees at two large office workplaces in Copenhagen, Denmark, and 653 (60%) replied (mean: age, 43 years; body mass index, 24 kg·m^−2^; and computer use, 90% of work time, 27/73% men/women).

### 2.2. Questionnaire Survey

Participants rated average neck/shoulder pain during the last three months on a numerical rating scale from 0 to 10, where 0 is “no pain” and 10 is “worst imaginable pain.” The rating scale was horizontally oriented to represent a modified visual-analogue scale [14]. A drawing from the Nordic Questionnaire defined the neck/shoulder area [15].

Participants were asked to manually palpate the muscle between the neck and shoulder (i.e., the midportion of the upper trapezius muscle) using a light squeeze with the opposite hand as shown by a picture and to rate tenderness on a scale of “no tenderness,” “some tenderness,” and “severe tenderness.” The actual force of the squeeze was not measured, but a pilot test in our lab showed that a light squeeze corresponded to approximately 20 N for most individuals as validated by squeezing lightly on a scale. Further, typical squeeze time ranged from 4 to 6 seconds.

Participants were also asked whether they had hypertension, heart disease, stroke, spinal disorders, fibromyalgia, rheumatoid arthritis, or problems due to traumatic injury. Participants were defined as having chronic disease when replying yes to one or more of these questions.

### 2.3. Statistics

Neck/shoulder pain intensity and prevalence of “no,” “some,” and “severe” tenderness, respectively, of the trapezius muscle were plotted on an *x*-*y* axis, and associations were fitted with a 2nd order polynomial. Additionally, the odds ratio for tenderness as a function of neck/shoulder pain intensity was determined using cumulative logistic regression controlled for age, gender, and chronic disease (Proc Logistic of SAS version 9.2). Odds ratios (OR) and 95% confidence intervals (95% CI) were calculated with tenderness as the dependent variables and neck pain intensity, age, gender, and chronic disease were calculated as independent variables.

## 3. Results

Table 1 shows demographics and neck/shoulder pain intensity of the respondents. The prevalence of “no,” “some,” and “severe” tenderness of the trapezius muscle was 18%, 59%, and 23% in women and 51%, 42%, and 7% in men, respectively, with a significant gender-difference (chi-square, *P* < 0.0001). Participants with “no,” “some,” and “severe” tenderness of the trapezius muscle, respectively, rated their neck/shoulder pain intensity to 1.5 (SD 1.6), 3.8 (SD 2.0), and 5.7 (SD 1.9) for women and 1.4 (SD 1.4), 3.1 (SD 2.2), and 5.1 (SD 1.7) for men.

In the cumulative logistic regression controlled for age, gender, and chronic disease, for every unit increase in neck/shoulder pain intensity, the OR for one unit increase in tenderness was 1.86 (95% confidence interval 1.70 to 2.04). The OR's for the other variables were 1.57 (95% confidence interval 1.07 to 2.30) for chronic disease (reference = no chronic disease), 3.11 (95% confidence interval 2.10 to 4.60) for women (reference = men), and 0.99 for age (95% confidence interval 0.98 to 1.01, n.s.).

**Figure 1:**
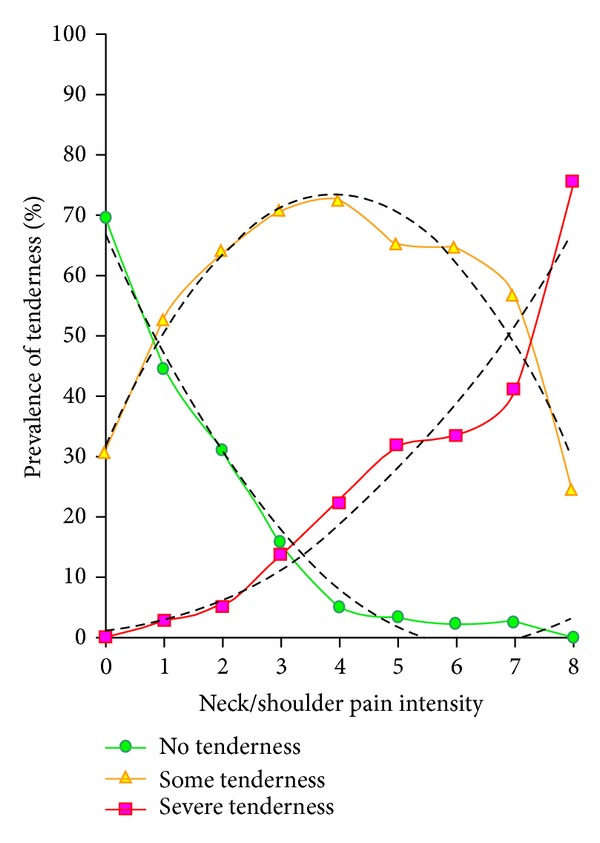
Neck/shoulder pain intensity (*x*-axis) and prevalence of no, some, and severe tenderness in the trapezius muscle (*y*-axis) among 653 office workers. The association could be almost perfectly fitted by a 2nd order polynomial (*R* ^*2*^ = 0.94–0.99).

Figure 1 shows that the association between neck/shoulder pain intensity and tenderness of the trapezius muscle could be almost perfectly fitted by a 2nd order polynomial (*R* ^*2*^ = 0.94–0.99) with the following equations where *y* equals the prevalence of trapezius muscle tenderness and* x* equals neck/shoulder pain intensity:

(1) yno  tenderness=1.7127x2−21.601x+66.931               (R2=0.9872),ysome  tenderness=−2.6846x2+21.181x+31.696                 (R2=0.9431),ysevere  tenderness=0.9723x2+0.4174x+1.3738                (R2=0.9424).

## 4. Discussion

Our study showed a strong association between perceived neck/shoulder pain intensity and trapezius muscle tenderness in office workers. The present study provides reference values of pain intensity among office workers with no, some, and severe tenderness of the trapezius muscle. The relevance of these findings is discussed below.

Our study shows a strong relationship between neck/shoulder pain and muscle tenderness. This confirms that the majority of neck/shoulder pain is related to myalgia, that is, pain and tenderness of the muscles [10, 12]. Juul-Kristensen and coworkers showed in a small study among 42 elderly computer workers with neck/shoulder pain that 38% had trapezius myalgia, 17% had tension neck syndrome, and 17% had cervicalgia [12]. Andersen and coworkers showed from clinical examination among 198 office workers with frequent neck/shoulder pain, from the total sample of 653 participants of the present study, that more than two-thirds experienced tenderness of the upper trapezius muscle [10].

The present study elaborates on these previous findings by providing reference values of pain intensity among office workers with no, some, and severe tenderness of the trapezius muscle. Thus, women and men with severe tenderness of the trapezius muscle had on average neck/shoulder pain intensities of 5.7 and 5.1, respectively. This knowledge can be used when defining relevant cut-points of pain intensity for inclusion of participants in future studies on neck/shoulder pain. Further, this knowledge can also be used to estimate the proportion of participants with muscle tenderness in studies that assessed only pain intensity, but not palpable tenderness. For instance, a study among the general working population with 1759 blue-collar and 3337 white-collar workers defined neck/shoulder pain cases as those having pain intensities of 4 or more [9]. According to the fitted 2nd order polynomial obtained from the *x*-*y* plot in Figure 1 of the present study, the expected prevalence of no, some, and severe tenderness among office workers with a neck/shoulder pain intensity of 4 is 7.9%, 73.5%, and 18.6%, respectively. Thus, it is likely that the majority of the white-collar workers in the previous study by Andersen had trapezius muscle tenderness.

The average differences in neck/shoulder pain intensity between no, some, and severe tenderness ranged between 1.7 and 2.3 on a scale of 0–10. Previous studies have argued that a difference in pain intensity of 1 on a scale of 0–10 is considered the minimally relevant difference in patients with chronic musculoskeletal pain, and a difference of 2 is considered to be moderately clinically meaningful [16]. Our study is in line with these findings by showing an approximate difference in pain intensity of 2 within each category on the tenderness scale of no, some, and severe tenderness.

Severe tenderness was more prevalent among women than men (23% versus 7%). This gender difference of tenderness is in line with findings in fibromyalgia patients [17], in workers with chronic neck/shoulder pain [10], in the hand muscles of healthy adults [18], and in the neck/shoulder muscles of healthy adults [19]. Although the exact reason for this gender difference remains unknown [20, 21], there are likely several influential mechanisms including differences in muscle size and testosterone levels. Thus, one study has shown a positive correlation between testosterone and pain thresholds; that is, higher testosterone was associated with less tenderness [22]. This may contribute to explaining the observed gender differences in the present and previous studies.

Our study has both strengths and limitations. The homogenous group of office workers reduces the risk of bias from socioeconomic confounding. However, this also limits the generalizability of our findings to office workers. Future studies should test the generalizability by including also workers with more strenuous work. Further, because all data were obtained from questionnaires, several sources of common method bias may influence the analyses. The most critical source is that information about tenderness and pain intensity has been delivered by the same person, that is, common-rater effects [23]. Controlling for chronic disease in the cumulative logistic regression analysis is a strength as it influences pain perception [24]. Thus, our study clearly shows that—even when controlling for chronic disease—there is a strong association between neck/shoulder pain intensity and trapezius muscle tenderness.

In conclusion, among office workers, a strong association between perceived neck/shoulder pain intensity and trapezius muscle tenderness exists. Further, tenderness was more common among women than men. The present study provides reference values of pain intensity among office workers with no, some, and severe tenderness of the trapezius muscle.

## Figures and Tables

**Table 1:** Demographics and neck/shoulder pain among all men and women of the study as well as men and women with no, some, and severe tenderness, respectively, in the trapezius muscle. The percentage of men and women with tenderness is provided in parentheses next to the number of participants in each category.

	All		No tenderness		Some tenderness		Severe tenderness	
	Men	Women	Men	Women	Men	Women	Men	Women
	*N* = 179	*N* = 474	*N* = 91 (51%)	*N* = 88 (18%)	*N* = 76 (42%)	*N* = 278 (59%)	*N* = 12 (7%)	*N* = 108 (23%)
Age (years), mean (SD)	43.5 (12.2)	42.7 (11.8)	43.9 (12.3)	44.9 (12.6)	43.2 (12.7)	41.7 (11.8)	41.9 (7.8)	43.7 (10.9)
Height (cm), mean (SD)	183 (7.0)	168 (6.2)	184 (6.6)	169 (6.0)	181 (7.4)	169 (6.0)	182 (5.9)	168 (6.7)
Weight (kg), mean (SD)	82.8 (10.5)	67.4 (12.1)	84.4 (9.6)	66.1 (11.5)	81.1 (11.1)	67.3 (12.6)	81.6 (12.0)	69.0 (11.0)
BMI kg (kg/m^2^), mean (SD)	24.8 (2.7)	23.8 (4.2)	24.9 (2.6)	23.3 (3.6)	24.6 (2.7)	23.6 (4.4)	24.8 (4.1)	24.6 (4.2)
Neck/shoulder pain intensity (0–10)	2.4 (2.1)	3.8 (2.3)	1.4 (1.4)	1.5 (1.6)	3.1 (2.2)	3.8 (2.0)	5.1 (1.7)	5.7 (1.9)
